# Potential Markers of Aggressive Behavior: The Fear of Other Persons' Laughter and Its Overlaps with Mental Disorders

**DOI:** 10.1371/journal.pone.0038088

**Published:** 2012-05-31

**Authors:** Elisabeth M. Weiss, Günter Schulter, H. Harald Freudenthaler, Ellen Hofer, Natascha Pichler, Ilona Papousek

**Affiliations:** Department of Psychology, Biological Psychology Unit, Karl-Franzens University, Graz, Austria; University of Medicine & Dentistry of NJ - New Jersey Medical School, United States of America

## Abstract

**Background:**

Anecdotal evidence suggested that some outbreaks of aggression and violence may be related to a fear of being laughed at and ridiculed. The present study examined the potential association of the fear of other persons' laughter (gelotophobia) with emotion-related deficits predisposing for aggression, anger and aggression proneness, and its overlaps with relevant mental disorders.

**Methodology/Principal Findings:**

Gelotophobic individuals were compared to a non-phobic control group with respect to emotion regulation skills and strategies, alexithymia, anger proneness, and aggressive behavior. Social phobia was diagnosed using the Structural Clinical Interview (SCID-I) for DSM IV (Diagnostic and Statistical Manual of Mental Disorders, Fourth Edition). Additionally, the SCID-II modules for Cluster A Personality Disorders, which includes schizoid, paranoid, and schizotypal personality disorder were administered to all participants. The findings show that gelotophobia is associated with deficits in the typical handling of an individual's own affective states, greater anger proneness and more aggressive behavior according to self-report as compared to non-phobic individuals. 80% of the subjects in the gelotophobia group had an additional diagnosis of social phobia and/or Cluster A personality disorder. The additional diagnoses did not predict additional variance of anger or aggressive behavior as compared to gelotophobia alone.

**Conclusions/Significance:**

Features related to aggression and violence that are inherent in mental disorders such as social phobia and Cluster A personality disorders may be particularly evident in the symptom of fear of other persons' laughter.

## Introduction

Aggression and violence pose a most difficult challenge to human welfare. Violence and crime in general have become worldwide public health problems, and highly publicized events in the US underscore intense public concern. Therefore, violence in the community has obvious social relevance for the political, criminal justice, and health care systems.

Aggressive and impulsive behavior that leads to criminal and antisocial acts may be the product of a failure of emotion regulation [1]. Healthy individuals are better at regulating their negative emotional states and benefit from restraint-producing environmental cues that also serve a regulatory role, such as facial and vocal signs of anger and fear. As accurate interpretation of facial expressions is important for social interaction, one would expect that individuals who have trouble interpreting facial expressions of emotions would be less socially competent and fail to adequately modulate behavior according to social context. Previous studies could show that aggressive individuals tend to generally interpret actions and intentions of others as involving anger and hostility [2], [3]. Additionally, they were more likely to perceive anger in emotionally neutral faces and show a negative emotional bias for ambiguous facial expressions [4]. Moreover, intimate partner violence perpetration has been related to a specific tendency to misperceive the partner's expressions of happiness as negatively valenced [5].

One important symptom, possibly related to outbreaks of aggression and violence, particularly in adolescents and young adults, may be “Gelotophobia” (from gelos, Greek for laughter), which is a young and still relatively unexplored construct. Gelotophobia is defined as the fear of other persons' laughter, meaning that individuals with gelotophobia connote laughter in their presence generally negatively and tend to assume that it is directed at them. They are hypervigilant towards signs of derision and persons that might ridicule them. They also tend to believe being strange by nature and to be strikingly emotionally inexpressive [6], [7]. Gelotophobia has attracted attention, because the pattern of emotion-related characteristics in gelotophobia seems to resemble those of violent individuals. Recent data suggested that individuals with higher levels of gelotophobia feel weak at downregulating their negative affect, and the attempts they typically make to manage their emotions are also considered inefficient by experts [8]. Moreover, they may be more likely to experience anger, also in emotionally neutral interpersonal situations [8], and have a tendency to recall interpersonal situations with a higher intensity of negative feelings [9]. Gelotophobia has also been related to being the victim of aggressive encounters. Experiences of being bullied or ridiculed initially were even suspected to cause or facilitate the development of gelotophobic symptoms [7], [10]. The suspicion that gelotophobia may be related to aggression and violence had recently been fuelled by anecdotal evidence suggesting that perpetrators of violent acts such as school shootings had a horror of being mocked and may have taken revenge for having been laughed at [11], [12].

However, the empirical indications of relationships of gelotophobia to emotion-related deficits and dispositions putatively relevant to aggressive behavior are preliminary in so far as they were obtained in studies that were correlational in nature, using convenience samples with a great majority of subjects having had low and sub-clinical levels of gelotophobia. Additionally, most studies did not control for psychiatric comorbidity. Like many other psychiatrically relevant symptoms, gelotophobia is considered to occur along a continuum in nonclinical populations, with levels exceeding a certain threshold considered as clinically relevant [12], [13]. Despite a growing number of scientific publications on gelotophobia, explorations of its relations to or overlaps with standard psychiatric diagnoses are still sparse. The most obvious overlap may be with social phobia, since both diagnoses share a preoccupation with fear of negative evaluation, humiliation, and embarrassment, a tendency to avoid social situations, and anxiety-related symptoms of physiological arousal [9]. However, some characteristics of gelotophobia may be not present in social phobia, that is, the emotional inexpressiveness, the belief to be strange and ridiculous by nature, and the threatening potential of every laughter, also from most familiar people and in all social situations [7], [9], [14].

Several features of gelotophobia that can be found in the case descriptions of Titze [7], such as the reduced emotional expressiveness, the belief of being strange by nature, and an attributional bias of the world as hostile and threatening, may suggest relationships to schizophrenia spectrum personality disorders. A first indication of potential overlaps was provided by a study indicating that psychiatric patients with schizophrenia and personality disorders scored higher on the standard gelotophobia instrument than other diagnostic groups such as mood and anxiety disorders [15]. However, no studies examining the prevalence of schizophrenia spectrum personality disorders in individuals classified as gelotophobic have been reported to date. The potential overlap of gelotophobia with schizophrenia spectrum personality disorders appears particularly likely when considering violence related issues and the proneness to anger and aggression. Several studies demonstrated that increased Cluster A personality disorder symptoms correlated significantly with violence [16], [17]. Especially delusions of “threat/control override” not only in mentally disordered subjects but also as a constituent of a paranoid personality style and referential style represent a significant risk factor for violence [18].

The primary goal of the present study was to examine the potential association between gelotophobia and anger and aggression proneness. In addition, overlaps with mental disorders that have been previously linked to gelotophobia (like social phobia) or provide a possible clinical risk for gelotophobia and violence such as Cluster A personality disorders were examined.

## Methods

### Ethics Statement

The study was performed in accordance with the 1964 Declaration of Helsinki and was approved by the Ethics Committee of the Karl-Franzens University, Graz. Written informed consent was obtained from all participants.

### Participants and Procedure

A total of 1440 university students from three local universities and a variety of disciplines (Biology, Business studies, Chemistry, Educational Sciences, Engineering, Geosciences, History, Language studies, Law, Medicine, Pharmacy, Psychology, Sociology, Theology) were screened using the standard diagnostic instrument for gelotophobia (Geloph<15>, [10]). Of these, 119 reached the cut-off score for gelotophobia (≥2.5), however, only 36 gelotophobics (26 women/10 men, aged 19 to 34 years, mean = 23.1, SD = 3.7) agreed to participate in the study. Additionally, 57 controls (scores<2.0), matched for age and study field were included. Four control subjects had to be excluded because of cannabis abuse, leading to a final sample of 53 non-phobic controls (28 women/25 men, aged 18 to 40 years, mean = 22.6, SD = 3.9). The mean Geloph<15> scores in the gelotophobia and the control group were mean = 2.8 (SD = .24) and mean = 1.2 (SD = .17), respectively. None of the non-phobic control group was taking psychoactive medication. Three participants in the gelotophobia group were taking antidepressives. Testing was conducted individually. After collection of demographic data, the participants were clinically interviewed and filled in the self-report scales. One test (the TEMT [19]) was administered in a separate test session one to three weeks apart. 19 gelotophobic and 20 non-phobic participants did not return to this second test session. (Additional data were obtained for purposes not relevant to the present research questions).

### Measures

#### Gelotophobia

The Geloph<15> [10] is a standardized self-report measure of gelotophobia including 15 items in a four-point answer format (1 “strongly disagree” to 4 “strongly agree”). The total score is calculated as the mean score of the 15 items. A sample item is “When others laugh in my presence I get suspicious”. Cut-off scores had been defined as following: 1.0–2.0: no gelotophobia; 2.0–2.5: borderline fearful; 2.5–3.0: slight expression of gelotophobia; 3.0–4.0: pronounced expression of gelotophobia. The distribution of scores of clinically diagnosed gelotophobics and the general population crosses at about 2.5 [12]. The Geloph<15> was originally developed in German and has also been psychometrically evaluated and validated in several other languages including English [20], Spanish [21], French [22], and Hebrew [23]. In the present study, the original German version was used [10]. Test reliability (Cronbach's alpha) was α = .94 in the present sample.

#### Emotion regulation

The emotion regulation subscale of the Self-report Emotional Ability Scale (SEAS [19]) assesses how able one feels to downregulate negative affect in everyday life and includes 6 items, which are rated on a six-point Likert scale (e.g., “When I'm scared of something I barely can't do anything about it”, “It's easy for me to get over a disappointing experience”). In the Typical-performance Emotional Management Test (TEMT [19]), 18 short descriptions of emotional situations are presented, followed by four response alternatives. Participants choose the alternative that best describes their typical behavior in the given situation. For each situation, the adequacy of the four behavioral alternatives had been determined by a panel of ten experts in the field who independently from each other had rated them from 1 to 4. The German version of the Emotion Regulation Questionnaire (ERQ [24]) was used to identify potential differences in the strategies used to handle emotional states. The ERQ comprises a subscale (4 items) on the habitual suppression of emotion-expressive behavior, that is, the tendency to not show one's emotions. The second subscale (6 items) assesses the disposition to use cognitive reappraisal. The items are rated on a seven-point Likert scale. Alexithymia was assessed with the German translation of the Toronto Alexithymia Scale (TAS-26 [25]). Alexithymia includes difficulties identifying and communicating one's feelings, and an externally oriented cognitive style with a relative lack of introspection [26]. It has been suggested that having difficulties to adequately perceive one's emotions may hamper the regulation of affect [27]. TAS scores can range from 18 to 90.

#### Anger

For the assessment of an individual's propensity to experience anger, the German version of the Spielberger State-Trait Anger Expression Inventory was used (STAXI [28], trait anger subscale, 10 items). Additional scales of the STAXI measure the tendency to direct anger inward and withhold expressions of angry feelings (anger-in subscale, 8 items), and the tendency to aggressively express anger towards other people or objects verbally or physically (anger-out subscale, 8 items). The items of the STAXI are rated on a four-point Likert scale.

#### Aggressive Behavior

Aggression proneness was assessed by the scales developed by Little et al. [29] to measure overt aggression in adolescents. Six items each are used to assess “pure” overt aggression (e.g., “I'm the kind of person who says mean things to others”), reactive overt aggression (e.g., “If others have angered me, I often hit, kick or punch them”), and instrumental overt aggression (e.g., “I often threaten others to get what I want”). Six items were used to measure victimization [30] (e.g., “I am the kind of person who is often put down by others”). The participants rated how true each item was for them on a four-point Likert scale from “not at all true” to “completely true”. None of the items in these scales referred to laughter as an aggressive act.

### Psychiatric Diagnosis

Social phobia was diagnosed using the Structured Clinical Interview for DSM-IV Axis I Disorders (SCID-I) [31]. Additionally, the Structured Clinical Interview for DSM-IV Axis II Personality Disorders (SCID-II) modules for Cluster A personality disorders including schizoid, paranoid, and schizotypal personality disorder were administered to all participants [32].

### Statistical Analysis

Differences between the gelotophobia vs. the non-phobic group were investigated using t-tests corrected for inequality of variances, if necessary. Scores on all dependent measures were symmetrically distributed except for the aggressive behavior scales, which were positively skewed. Consequently, results for the aggressive behavior scales were confirmed by nonparametric (Mann-Whitney U) tests. As correlations may be overestimated because of group differences in central tendency, intercorrelations among the dependent variables were calculated using partial correlations controlling for group (gelotophobia vs. non-phobic group). Differences between groups according to psychiatric diagnoses were investigated using the Kruskal-Wallis test, because these group sizes were not large enough for the central limit theorem to take effect. A two-tailed significance level of p<.05 was used for all analyses.

## Results

### Emotion Regulation

Participants in the gelotophobia group described themselves as less able to regulate their negative emotions than their non-phobic counterparts (SEAS; t = −8.6, df = 87, p<.001), and their typical approaches to manage their emotions were less efficient as judged by experts (TEMT intrapersonal scale; t = −3.0, df = 22.2, p<.01). Participants with gelotophobia indicated a stronger tendency to not show their emotions than participants in the control group did (ERQ suppression subscale; t = 4.9, df = 87, p<.001), but there was no significant difference in the use of cognitive reappraisal (t = −1.7, df = 87, ns.). Finally, alexithymia scores were higher in the gelotophobia than in the control group (t = 6.0, df = 87, p<.001). Means and standard deviations are shown in Table 1. Table 2 shows the intercorrelations among the emotion regulation variables. To exclude the possibility that differences may be influenced by the different gender compositions of the two groups, the analyses were re-run comparing only the female participants of the gelotophobia (N = 26) and the control group (N = 28). The pattern of differences remained the same (SEAS t = −6.9, df = 52, p<.001; TEMT t = −2.2, df = 14.3, p<.05; ERQ suppression t = 3.7, df = 52, p<.001; ERQ reappraisal t = −1.7, df = 52, ns.; TAS t = 5.1, df = 52, p<.001).

**Table 1:** Differences Between Gelotophobics and Non-phobic Controls: Emotion Regulation.

	Gelotophobia groupN = 36(Mean ± SD)	Control groupN = 53(Mean ± SD)	
SEAS Emotion regulation	17.9±4.4	26.0±4.4	p = .000
TEMT Intrapersonal management*	53.5±8.2	59.8±4.9	p = .007
ERQ Suppression	15.6±4.6	11.1±4.0	p = .000
ERQ Reappraisal	27.7±5.1	29.8±6.1	p = .099
TAS Alexithymia	47.0±8.4	37.3±6.8	p = .000

*Note*.

* For the TEMT, N = 17/33.

**Table 2:** Intercorrelations Among Emotion Regulation Variables.

	(2)	(3)	(4)	(5)
(1) SEAS Emotion regulation	.38**	.08	.35***	−.29**
(2) TEMT Intrapersonal management		−.22	.31*	−.26
(3) ERQ Suppression			.04	.18
(4) ERQ Reappraisal				−.19
(5) TAS Alexithymia				

*Note*.

* p<.05,

** p<.01,

*** p<.005;

SEAS, ERQ, TAS: N = 89; TEMT: N = 50.

### Anger

Gelotophobics showed a greater general propensity to experience anger than participants in the control group (STAXI trait anger; t = 5.6, df = 54.4, p<.001). The increased anger proneness in gelotophobia is reflected in higher levels of anger that is directed inwards (STAXI anger in; t = 5.7, df = 47.9, p<.001) as well as in anger that is directed towards other people or objects (STAXI anger out; t = 3.3, df = 86, p<.005). Means and standard deviations are shown in Table 3. Intercorrelations among the anger scales were: trait anger×anger in r = .30, p = .005; trait anger×anger out r = .68, p<.001; anger in×anger out r = .16; ns. (partial correlations controlling for group). The pattern of results did not change when only female participants were compared (trait anger t = 5.1, df = 38.3, p<.001; anger in t = 4.6, df = 37.1, p<.001; anger out t = 2.5, df = 51, p<.05).

**Table 3:** Differences Between Gelotophobics and Non-phobic Controls: Anger, Aggressive Behavior and Victimization.

	Gelotophobia groupN = 36(Mean ± SD)	Control groupN = 53(Mean ± SD)	
STAXI Trait anger	21.3±5.7	15.3±3.6	p = .000
STAXI Anger in	17.8±5.6	12.0±2.9	p = .000
STAXI Anger out	13.7±3.8	11.2±3.1	p = .002
“Pure” overt aggression	8.3±1.3	7.0±1.3	p = .003
Reactive overt aggression	9.8±2.3	8.7±2.4	p = .016
Instrumental overt aggression	7.7±1.9	6.6±0.9	p = .004
Victimization	8.6±2.1	6.5±1.0	p = .000

*Note*. Trait anger: general propensity to experience anger; Anger in: tendency to direct anger inward and withhold expressions of angry feelings; Anger out: tendency to aggressively express anger towards other people or objects verbally or physically.

### Aggressive Behavior

Participants in the gelotophobia group characterized themselves as more prone to aggressive behavior than participants in the control group did. This held for all three types of overt aggression (“pure” overt t = 3.0, df = 52.7, p<.005; reactive t = 2.4, df = 87, p<.05; instrumental t = 2.9, df = 45.6, p<.005). In addition, gelotophobics indicated more often being the victim of aggressive behavior than their non-phobic counterparts (t = 5.6, df = 46.8, p<.001). Means and standard deviations are shown in Table 3. Nonparametric Mann-Whitney U tests yielded identical results (“pure” overt z = 3.0, p<.005; reactive z = 2.4, p<.05; instrumental z = 2.9, p<.005; victimization z = 5.6, p<.001). In women-only comparisons the pattern of results remained the same, except for reactive aggression which fell below the significance threshold (“pure” overt t = 3.3, df = 31.9, p<.005; reactive t = 1.7, df = 52, p = .09; instrumental *t* = 2.8, df = 29.7, p<.01; victimization *t* = 5.3, df = 30.1, p<.001). For intercorrelations among the aggression scales see Table 4.

**Table 4:** Intercorrelations Among Aggression Variables.

	(2)	(3)	(4)
(1) “Pure” overt aggression	.30***	.71***	.19
(2) Reactive overt aggression		.34***	.13
(3) Instrumental overt aggression			.15
(4) Victimization			

*Note*.

* p<.05,

** p<.01,

*** p<.005;

N = 89.

### Psychiatric comorbidity for social phobia and Cluster A personality disorders

**Figure 1:**
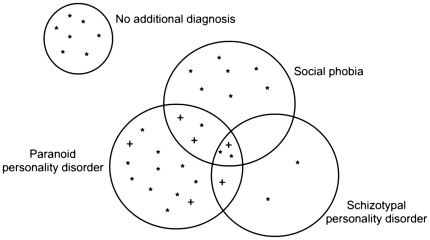
Psychiatric comorbidity for social phobia and Cluster A personality disorders among participants in the gelotophobia group. *Note.* Each symbol represents one participant; plus signs denote participants with an additional diagnosis of schizoid personality disorder.

Figure 1 shows the diagnoses of social phobia and Cluster A personality disorder and their overlaps among participants in the gelotophobia group. In the control group, two participants were diagnosed with social phobia and one participant with paranoid personality disorder. 80% of the participants in the gelotophobia group had an additional diagnosis of social phobia and/or Cluster A personality disorder (social phobia N = 7, Cluster A personality disorder N = 15, combined social phobia and Cluster A diagnosis N = 7).

The group of gelotophobics carrying no additional psychiatric diagnosis (N = 7) and the gelotophobic groups with psychiatric comorbidity did not differ in their Geloph<15> scores (χ^2^ = 4.8, df = 3, ns.). Neither did the analysis of the emotion regulation and anger scales and the scales concerning overt aggressive behavior reveal any significant differences among these groups. That is, within the gelotophobia group the psychiatric diagnoses did not explain any additional variance of aggressive behavior. Only on the victimization scale scores were highest in the combined social phobia plus Cluster A personality disorders group (mean = 10.9, SD = 1.9) compared to the gelotophobia only (mean = 7.6, SD = 1.7), social phobia (mean = 8.3, SD = 2.2), and Cluster A groups (mean = 8.1, SD = 1.7; χ^2^ = 9.4, df = 3, p<.05).

## Discussion

Inspired by anecdotal evidence that some outbreaks of aggression and violence may be related to a fear of being laughed at and ridiculed, the present study examined the potential association between gelotophobia and anger and aggression proneness. In addition, overlaps with mental disorders that have been previously linked to gelotophobia (like social phobia) or provide a possible clinical risk for gelotophobia and violence such as personality disorders were examined.

The findings show that gelotophobia is associated with several deficits in the typical handling of an individual's own affective states. Gelotophobic participants indicated that they felt weak at regulating their emotions to a greater extent than non-phobic controls did. Moreover, their typical approaches to manage their emotions in emotion-laden situations are considered inefficient by experts. The indication of less efficient strategies in the management of affective states is further corroborated by the finding that gelotophobics more often try to handle their emotions by suppressing emotion-expressive behavior than their non-phobic counterparts. By contrast, no differences were found for the use of cognitive reappraisal. These results directly replicate findings of previous studies that were obtained in the lower, sub-clinical range of gelotophobia using it as a continuous variable [8] and are in agreement with observations from clinical encounters that gelotophobics are anxious to maintain an inconspicuous appearance [7]. As expressive suppression involves the inhibition of only the behavioral component of one's emotional response, it is generally regarded an inefficient strategy for the downregulation of negative feelings [33]. Deficits in the perception of one's emotions (indicated by high alexithymia scores [26]) may hinder their adequate handling [27].

Dysfunction in basic emotion regulation processes are assumed to predispose to aggression and violence [1]. More specifically, deficient ability to identify one's emotional state (as assessed by the TAS) was linked to aggression in psychiatric patients [34]. Emotional inexpressivity may further contribute to aggressive behavior insofar as a failure to express emotions in a healthy way leads to a reliance on maladaptive ways of expressing emotions, such as through verbal and physical aggression [35].

Previous experimental results had suggested that anger may show a certain predominance in gelotophobics when they are involved in interpersonal situations [8]. This was confirmed by the present finding of higher levels of trait anger in the gelotophobia than in the non-phobic control group. Trait anger plays a role in aggressive behavior and violence, particularly when coupled with defective emotion regulation [36], [37].

Finally, the findings also more directly indicated a link between gelotophobia and aggression, showing a stronger tendency towards overt aggression in gelotophobics than in non-phobic controls, at least according to self-report. The findings were similar for “pure”, reactive, and instrumental aggression (except that in women the difference in reactive aggression was weaker and fell below the significance threshold). Participants in the gelotophobia group also indicated being the victim of aggressive encounters to a greater degree than their non-phobic counterparts. At the first glance, this may well fit the fear of being laughed at and ridiculed. Initially, it was hypothesized that experiences of being bullied or ridiculed may cause or facilitate the development of gelotophobic symptoms [7], [12], but gelotophobia could not be traced back to repeated or intense experiences of having been laughed at and ridiculed in childhood and youth [13]. Instead, it has been proposed that gelotophobics may overestimate incidents of having been bullied, because they misinterpret harmless comments as offensive [20]. The latter interpretation may be in accordance with the overlap of gelotophobic symptoms with schizophrenia spectrum personality disorders (particularly paranoid symptoms).

One aim of the study was to further assess the overlap between gelotophobia and other mental disorders, especially schizophrenia spectrum personality disorders and social phobia. Nestor et al. [18] defined four fundamental personality dimensions that increase the risk for violence and may be specifically important as clinical risk factors among persons with mental disorders such as schizophrenia and personality disorders. The first two dimensions relate to deficits in the regulatory functions of impulse control and affect regulation, which are core deficits in all mental disorders and can also clearly be seen in our group of gelotophobics. The last two dimensions relate to personality surface traits such as narcissistic injury (threatened egotism) and paranoid cognitive personality style, which elevate the rates of violence especially in schizophrenia spectrum disorders. The current study showed that 80% of the subjects in the gelotophobia group had an additional diagnosis of social phobia and/or Cluster A personality disorder, in which these dimensions are inherent.

Interestingly, the classification into Cluster A personality disorder and/or social phobia did not show significant differences regarding anger-proneness or aggressive behavior among the sub-groups within the gelotophobia group. That is, none of these diagnoses did explain additional variance of overt aggressive behavior as compared to gelotophobia alone. However, a higher victimization score was observed in the combined social phobia plus Cluster A personality disorders group. In a recent study by Raine et al. [38] the relationship between schizotypal personality and aggression in children was mediated by peer victimization. Typical symptoms of Cluster A personality disorders and social phobia such as social anxiety, paranoid ideation, blunted affect, and odd behavior can easily lead to victimization in children and finally result in reactive aggression as a defensive response to provocation [39]. However, due to the potential misinterpretation of social situations in individuals with schizophrenia spectrum personality disorders and gelotophobia, it would be necessary to use peer ratings of victimization (instead of self-report) in order to reliably test the model proposed by Raine et al. [38] of a viscous cycle of schizotypal features and victimization that may eventually erupt into violence.

Being the first study examining the interrelationships between gelotophobia, schizophrenia spectrum personality disorders, and clinically diagnosed social phobia, the study needs to be interpreted with caution, mainly because the sample size is limited and all participants were university students. Therefore, the results may not generalize to other populations.

Aggression is closely linked to hypervigilance towards stimuli that could be perceived as threatening [39], [40]. This feature which is inherent in social anxiety and paranoid ideation may be particularly evident in the fear of other persons' laughter. Taken together, therefore, gelotophobia could be a core symptom for aggressive behavior underlying different mental disorders such as social phobia or Cluster A personality disorders.
